# Survival of Microorganisms on Filtering Respiratory Protective Devices Used at Agricultural Facilities

**DOI:** 10.3390/ijerph16162819

**Published:** 2019-08-07

**Authors:** Anita Jachowicz, Katarzyna Majchrzycka, Justyna Szulc, Małgorzata Okrasa, Beata Gutarowska

**Affiliations:** 1 Institute of Fermentation Technology and Microbiology, Lodz University of Technology, Wólczańska 171/173, 90-924 Łódź, Poland; 2 Department of Personal Protective Equipment, Central Institute for Labour Protection—National Research Institute, Wierzbowa 48, 90-133 Łódź, Poland

**Keywords:** microorganisms, filtering facepiece respirators (FFRs), workplaces, dust

## Abstract

Bioaerosol is a threat at workplaces, therefore the selection and safe use of filtering facepiece respirators (FFRs) is important in preventive activities. The aim of the study was to assess the survival of microorganisms on materials used for FFRs construction. The parameters for microorganism growth under model conditions were described using the Gompertz equation, model verification was also carried out using FFRs at the farmers’ workplaces. We found that the factors determining a high survival of microorganisms were as follows: moisture corresponding to the conditions of use and storage of FFRs at workplaces, the presence of sweat and organic dust; inorganic dust and addition of biocide in nonwovens limited the growth of microorganisms, resulting in a shortening of the stationary growth phase and decreased cell numbers (5–6 log). Dust concentration at workplaces was higher than EU occupational exposure limit values and WHO recommendations for airborne particulate matter. Microbial contaminations of the air (10^3^–10^4^ CFU/m^3^), settled dust (10^4^–10^6^ CFU/g) and FFRs (10^5^ CFU/4cm^2^) during the grain harvest were high, the main contamination being bacteria (actinomycetes, *Pseudomonas fluorescens*) and xerophilic fungi. A high correlation was found between the number of microorganisms and the weight of dust on FFRs (R^2^ = 0.93–0.96).

## 1. Introduction

The main group of harmful biological factors occurring in workplaces are allergenic and toxic agents forming bioaerosols. Bioaerosols are biological particles of organic and inorganic dust and biological fractions suspended in the air, including viruses, bacteria, endotoxins, fungi and secondary metabolites of fungi, as well as particles of plant and animal origin [1,2,3]. After entering the respiratory system, these factors can cause irritative, toxic, allergic, carcinogenic or fibrotic reactions, resulting in diseases such as chronic obstructive lung disease, bronchial asthma, chronic bronchitis, bronchial hyperresponsiveness and organic dust toxic syndrome. Furthermore, they can irritate mucous membranes, skin and conjunctivae [4,5].

Bioaerosols present serious health problems for agriculture, cheese factories, the wood industry, composting plants, waste sorting plants, poultry farms, medical and veterinary facilities, diagnostic laboratories, plants producing biofuel, libraries, and art facilities [6,7,8,9,10,11,12]. The next-generation sequencing of bioaerosols emitted, in a waste sorting plant, showed a higher biodiversity than that previously reported for this working environment in studies carried out using culture methods followed by the identification of microorganisms [13]. It is probable that high-throughput sequencing technology will detect a higher number of harmful biological agents in other workplaces as well. Therefore, in these working environments, it is recommended that personal protective equipment (PPE) be used, particularly disposable and reusable filtering facepiece respirators (FFRs) [14].

The workplace protection factor (WPF) is commonly used to assess respirator performance in the workplace. It is defined as a ratio of the particle concentration outside the respirator to that inside the respirator, and is a measure of the protection provided in the workplace under the specific conditions. Lee et al. (2005a; 2005b) developed a new personal sampling system and used it in laboratory and field studies, including to determine the WPF provided to farmers by N95 filtering facepiece respirators against airborne dust and microorganisms of different particle size range [15,16].

More than 50% of the WPFs for microorganisms (mean aerodynamic diameter <5 μm) were less than the proposed recommended assigned protection factor (APF) of 10. Therefore, authors claim N95 filtering facepiece respirators seem inadequate against microorganisms in agricultural working environments [15,16].

For several years, intensive work has been underway on modifying FFRs materials and also developing new forms of FFRs that would have antimicrobial properties. Zheng et al. (2016) enhanced the nanosilver-coating ability of nonwoven fabrics from a particulate respirator through surface modification by sodium oleate (the surfactant improved the fabrics’ water wet preference). Developed by the authors, functionalized particulate respirators with silver nanoparticles had excellent antimicrobial activities against *Staphylococcus aureus* and *Pseudomonas aeruginosa* [17].

In turn, Park et al. (2018) demonstrated the feasibility of using microorganism-ionizing respirators with reduced breathing resistance to remove airborne bacteria by demonstrating the feasibility of using microorganism-ionizing respirators with efficiencies of 75–100% against *Escherichia coli*. They claim that respirators with miniaturized corona ionizers and two pairs of separator electrodes are safe and can be useful for healthcare workers during an airborne pathogenic outbreak and for patients with compromised breathing capacity [18].

Currently, because of lower operating costs and reduced waste generation, reusable FFRs are becoming increasingly popular. During use, the exhalation of humid air and sweat increases the moisture content in the filter material. Lin et al. (2017) investigated the effects of artificial saliva, artificial perspiration and storage conditions on the survival of bioaerosols and the filter performance of FFRs. They showed that artificial saliva and artificial perspiration increased the survival of *Bacillus subtilis* in N95 masks and in surgical masks [19]. This, together with the organic and inorganic substances deposited from the environment during air filtration, create optimal conditions for the growth of microorganisms [20]. Many studies have confirmed the possibility of the growth of both bacteria and fungi on filter material [21,22,23,24,25,26]. Perrier et al. (2008) tested the growth of microorganisms from the activated sludge of a wastewater treatment plant and a toluene specific consortium onto filter media and their ability to proliferate onto filter media. Using a static growth procedure during 10 days under 100% relative humidity, artificially contaminated filters were submitted to microbial colonisation. They indicated that the colonised filters released up to 4.50 × 10^2^ microbial particles/cm^3^ and the soot may thus have played an interface role in microbial adhesion onto the filters [27]. Nevertheless, little is known about the impact of environmental conditions in which FFRs can be used, i.e., the kinds of microorganisms, humidity variables and temperature conditions, contents and types of dust. Also, the impact of the presence of sweat or biocide in filtering nonwovens on the survival of microorganisms on the various filtering materials used for the respiratory protection of workers is not entirely clear. Such knowledge is essential for the proper selection of FFRs and the timing of their use, depending on the characteristics of the specific workplaces.

In order to describe the influence of various factors on the survival of microorganisms in the environment, probabilistic, kinetic, primary, secondary and tertiary mathematical models were used. The most common one in predictive microbiology is the Gompertz equation, which describes the dependence of the number of microorganisms on time [28]. However, there is a lack of research on the use of mathematical models to describe the survival of microorganisms depending on environmental factors that occur during the use of FFRs at workplaces.

Therefore, the aim of this study was to assess the impact of environmental factors (variable humidity and temperature, presence and concentration of organic and inorganic settled dust, acid and alkaline sweats, presence of biocide in nonwoven) on the survival of microorganisms on filtering materials used for the construction of FFRs. Parameters of microorganism growth under model conditions were described using the Gompertz equation. Verification of the obtained relationships was also performed in conditions of FFRs use at farmers’ workplaces.

## 2. Materials and Methods

### 2.1. Model Study

#### 2.1.1. Filtering Nonwovens

Two types of filtering nonwovens were studied: (1) pristine Polypropylene nonwoven (control sample) and (2) composite polypropylene nonwoven containing biocide (TH2227, Sanitized AG, Burgdorf, Switzerland, 2 g per 0.37 m^2^ nonwoven sheet) and water-absorbing additive (EK-X EN52, Nippon/Ecotec, 1 g per 0.37 m^2^ nonwoven sheet). Both types were manufactured using melt-blown technology. All of the technological works were carried out on an experimental stand for the production of electret melt-blown nonwovens, which was described in detail in [29]. Compressed air was used in order to inject the modifier directly into the stream of semi-solidified fibers that were leaving the spinning head during nonwoven production. It was made possible by the use of a specially designed device that was mounted inside the spinning head. The device was connected to the rotary dispenser from which the modifier was added symmetrically to the fiber-forming zone without interaction with the heating zones of the device, thus ensuring their full functionality [30,31]. The nonwovens were corona-charged during production to enhance their filtering properties. The characteristics of the selected nonwovens are shown in Table 1.

High efficiency melt-blown electret nonwovens constitute the most important layer in the construction of FFRs because they are responsible for the filtration of fine dust particles from the airflow. Usually pristine nonwovens (such as nonwoven no. 1, Table 1) are used by the manufacturers, but recent literature points to the fact that, in case of FFRs protecting against biological agents, the biocidal and moisture absorbing properties of those materials (such as in the case of nonwoven no. 2, Table 1) would contribute to the safety of workers [31].

Filtering nonwoven swatches of the surface area of 4 cm^2^ were prepared for the studies and were disinfected under a UV-lamp for 3 h.

All of the factors considered within the study (i.e., the influence of variable humidity and temperature, the presence and concentration of organic and inorganic settled dust and acid and alkaline sweats) were tested using nonwoven no. 1 (Table 1) that was subjected to different kinds of preconditioning (as described further in Section 2.1.3, Section 2.1.4 and Section 2.1.5). The influence of the presence of biocide in the nonwoven swatches was determined by comparing the control sample (pristine sample of nonwoven no. 1) with the nonwoven containing a biocidal agent (nonwoven no. 2 without preconditioning).

#### 2.1.2. Microorganisms

Five microorganism species were obtained from the American Type Culture Collection (ATCC) and the National Collection of Agricultural and Industrial Microorganisms (NCAIM), including: *Bacillus subtilis* (NCAIM 01644), *Escherichia coli* (ATCC 10536) and *Staphylococcus aureus* (ATCC 6538) bacteria, *Candida albicans* (ATCC 10231) yeast and *Aspergillus niger* (ATCC 16404) mould, were used for the studies.

#### 2.1.3. Thermal and Humidity Conditioning

**Figure 1:**
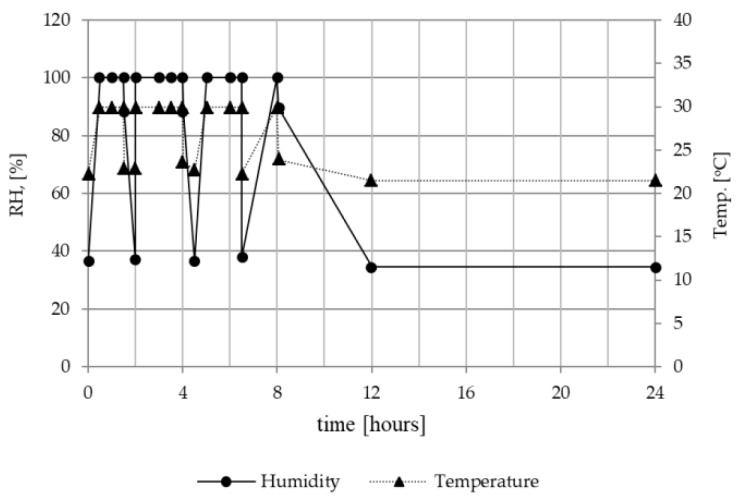
Daily changes of temperature and air humidity used for conditioning of filtering nonwovens.

Temperature and relative humidity (RH) levels selected for the conditioning of the nonwoven samples were established based on the results of measurements simulating FFRs use at the workplace presented in [20]. A 24-h cycle shown in Figure 1 corresponded to an 8-h work shift and 16-h storage period. Nonwoven swatches were subjected to thermal conditioning in a climatic chamber (Binder-720, Tuttlingen, Germany) simulating filtering facepiece respirator (FFRs) use conditions. The samples were placed in the chamber at a temperature of 30 ± 1 °C and RH of 100% ± 1% for 8 h. After 1.5, 4 and 6 h, a break in work lasting 30 min was simulated by taking the samples out of the chamber and storing them in normal conditions (22 ± 1 °C; 38% ± 1%). After 8 h, samples were removed and stored in normal conditions (22 ± 1 °C; 38% ± 1%) for the remaining 16 h. Thermal conditioning cycle was repeated from nonwoven samples incubated for longer than 24 h.

#### 2.1.4. Deposition of Organic and Inorganic Dust on Filtering Nonwovens

Two types of dust were deposited onto filtering nonwovens: Organic dust from a composting plant (Łódź, Poland) collected at a refuse homogenization hall, and inorganic dust from a cement plant (Chełm, Poland) collected from a clinker conveyer belt. Detailed chemical, microbiological, granulometric and toxicological characteristics were presented in an earlier publication [36]. The research showed that at workplaces in composting and cement plants, the dominant PM fraction had an aerodynamic diameter below 1 μm, accounting for 87.3% and 80.8% of the total dust concentration. The cement plant dust was characterised by a low carbon to nitrogen ratio (C:N about 10), and the composting dust by a high ratio of carbon to nitrogen (C:N = 98.64). Dust from cement plant was alkaline, dust from composting plant were slightly acidic [36]. Filtering nonwoven samples of 79 cm^2^ were prepared according to methods described by Majchrzycka et al. [26]. Dust was deposited onto the nonwovens in a dust chamber (Thomex-MB type, MedBryt, Poland) at a fixed air flow rate (95 L/min) through filtering materials. Deposition times (2 and 4 min) were set such that the mass of deposited dust corresponded to the mass of dust retained on the filtering FFRs layers during work hours at workplaces with medium and high dust pollution. Dust samples were sterilized at 115 °C for 15 min and dried for 24 h at 70 °C under the reduced pressure of 100 mbar in a drying chamber (VD 53, Binder, Germany). The correlations between the amount of dust deposited on nonwovens, deposition times and dust types are shown in Table 2.

#### 2.1.5. Acidic and Alkaline Sweat

One-hundred microliters of freshly prepared acidic or alkaline sweat solution were placed onto filtering nonwovens, then samples were examined following the procedure described in Section 2.1.6. Sweat solutions were prepared according to the PN-EN ISO 105-E04:1996 norm.

#### 2.1.6. Assessment of Microorganism Survival on Filtering Nonwovens

The AATCC 100-1998 “Antimicrobial Finishes of Textile Materials” quantitative method was used to assess microorganism survival on filtering nonwovens. Microbial inocula were prepared according to a previously described method [37]. The inoculation suspension density at the level of 1.9 × 10^6^ CFU/mL for *B. subtilis*, 7.5 × 10^8^ CFU/mL for *E. coli*, 8.7 × 10^9^ CFU/mL for *S. aureus*, 1.0 × 10^8^ CFU/mL for *C. albicans* and 3.8 × 10^6^ CFU/mL for *A. niger* was determined using the culture method and Thom chamber. Filtering nonwoven swatches (4 cm^2^) were seeded with 25 µL of inoculation suspension and incubated in a climatic chamber (Binder-720, Tuttlingen, Germany) at 30 ± 2 °C and RH of 80% (except for conditions described in Section 2.1.3). Nonwoven samples were tested immediately post-inoculation (at *t* = 0 h) and subsequently at 2, 3, 4, 6, 8, 12, 24, 48, 72, 96 and 120 h of incubation. Sample incubation time for the studied bacteria was a maximum of 96 h in total, while the incubation of samples for fungi was extended to 120 h because of their growth physiology. Following incubation, nonwovens were placed into 50 mL of sterile 0.85% NaCl saline solution and shaken for 5 min. Next, serial dilutions from 10^−2^ to 10^−6^ were performed and 1 or 0.1 mL of dilutions were plated onto sterile Petri dishes. Tryptic Soy Agar (TSA, Merck, Darmstadt, Germany) or Malt Extract Agar (MEA, Merck, Germany) semisolid medium was poured over for bacteria and fungi, respectively. The plates were incubated at 30 ± 2 °C for 24/48 h (bacteria/fungi) and at 27 ± 2 °C for 72 h (fungi). The colonies grown were counted and the result expressed as CFU/sample. Tests on nonwovens were performed in three independent replicas for each experiment (time, microorganism type, studied factor).

### 2.2. Study at Workplaces

#### 2.2.1. Filtering Facepiece Respirators

We used single-use FFRs of the FFP3 class according to EN 149:2001 [38], intended to protect the respiratory system against dust, particulate matter and liquid aerosol. The foldable face part was made of three layers of polypropylene electret (PPQ) filtrating nonwoven covered with a external spun-bonded nonwoven. The FFRs were equipped with the nasal seal, improving tightness and a valve lowering exhalation resistance and helping with removal of excess of water vapor and carbon dioxide from the breathing zone. The service lives of FFRs at the tested workplaces were 15, 30, 60, 90 and 120 min. The FFRs were weighed before and after the tests to determine the mass of retained dust (R160P laboratory balance, Sartorius GMBH, Göttingen, Germany).

#### 2.2.2. Ambient Conditions at the Workplace

The studies were conducted in July 2018 at two farms during the wheat harvest. Temperature, RH, air flow velocity, air and FFRs pollution level, microbiological pollution level of air, sedimented dust and FFRs during work hours were measured. All measurements were performed at 1.5 m from the ground/floor level. Temperature, RH and air flow velocity were measured using a VelociCalc^®^ Multi-Function Velocity Meter 9545 (TSI, Shoreview, MN, USA). Microclimate measurements for every test variant were conducted in triplicate for 1 min every 5 s. Workplace characteristics are given in Table 3.

#### 2.2.3. Analysis of Dust Fractions Suspended in the Air

The concentration of airborne dust was measured using a DustTrak™ DRX Aerosol Monitor 8533 (TSI, Shoreview, MN, USA) portable laser photometer. It allowed for the simultaneous measurement of mass concentrations of PM_1_, PM_2.5_, PM_4_ and PM_10_ dust fractions and total dust mass concentration. The measurements were conducted continuously at every site with a sampling interval of 5 s. The analysis was performed prior (X) and during work (Y) at studied workplaces.

#### 2.2.4. Microbiological Analysis of Air, Sedimented Dust and FFRs

Sedimented dust samples were collected in sterile containers during the entire work cycle at workplaces. Collected dust samples were stirred with a sterile spoon. Six random 1 g samples were taken, poured into sterile containers and mixed. Thus, 0.1 g samples were taken for microbiological analyses in triplicate. Samples were diluted in sterile saline solution and pour-plated with the scope of determination as follows: Overall fungi number (MEA medium with 0.1% chloramphenicol); xerophilic fungi number (DG18 LAB-AGAR™, Biocorp, Warsaw, Poland); overall bacteria number (TSA with 0.2% nystatin), actinomycetes(Pochon Agar 0.2% nystatin, Labomix, Lodz, Poland); *Staphylococcus* sp., including mannitol-positive and-negative species (*Staphylococcus* Selective Agar acc. to Chapman, Merck, Germany), *Pseudomonas fluorescens* bacteria (Agar King B, Noack, Warsaw, Poland). Incubation conditions included: 37 ± 2 °C, 24–48 h (mannitol-positive and -negative *Staphylococci*), 25 ± 2 °C, 5–7 days (overall fungi number, xerophilic fungi and actinomycetes), 30 ± 2 °C, 48 h (overall bacteria number, *Pseudomonas fluorescens*). Following the appropriate incubation time, the colonies were counted and the results expressed as CFU/g of dust.

FFRs were collected following 15, 30, 60, 90 and 120 min of working time. Samples of filtering nonwoven fragments (4 cm^2^) in three replications were taken from the place most exposed to dust (filtration part). Then, the filtering nonwoven fragments of the known surface area deriving from FFRs were placed into 50 mL of saline solution and shaken for 5 min. Dilutions were made and plated onto TSA medium with 0.2% nystatin to determine the overall bacteria number or MEA with 0.1% chloramphenicol to determine overall fungi number. Plates were incubated at 30 ± 2 °C for 24 h (bacteria) or at 27 ± 2 °C for 72 h (fungi). Grown colonies were subsequently counted and the results were expressed as CFU/4 cm^2^. Microbiological analyses of FFRs were performed in duplicate at the combine harvester operator workplace.

#### 2.2.5. Mathematical and Statistical Calculations

The Gompertz model, described in formula 1, was used for the modelling of microorganism growth on filtering nonwovens using DMFit software (Version 3.5, Institute of Food Research, Norwich, UK).

(1) $$N\left(t\right)=A+Ce^{-e^{\left[-B\left(t-M\right)\right]}}$$

where N(t) is log of bacteria number over time *t*, *A* is the asymptote log of bacteria number at an undetermined time lapse (on average = initial bacteria number log), *C* is the asymptote of growth dimension that occurred at the undetermined time lapse (growth cycle number), *M* is maximal growth velocity time, and *B* is relative growth velocity over time *M*. Microorganism growth parameters: Stationary phase beginning time (t_stat_) and cell increment (Y_max_), were determined by the chosen model. Microorganism growth phases were established using Origin^®^ 6.1 software (OriginLab, Northampton, MA, USA). Microsoft^®^ Excel 2010 (Microsoft, Redmond, MA, USA) was used to calculate the arithmetic means and standard deviations of microorganism numbers on nonwoven samples. Statistical differences between the number of any given microorganism group for different dust or air types were determined using Origin^®^ 6.1 software. Finally, granulometric distribution analysis at workplaces was performed (univariate analysis of variance, ANOVA, significance level α = 0.05) using Statistica 13.1 (Statsoft, Tulsa, OK, USA).

## 3. Results

### 3.1. Model Study

**Figure 2:**
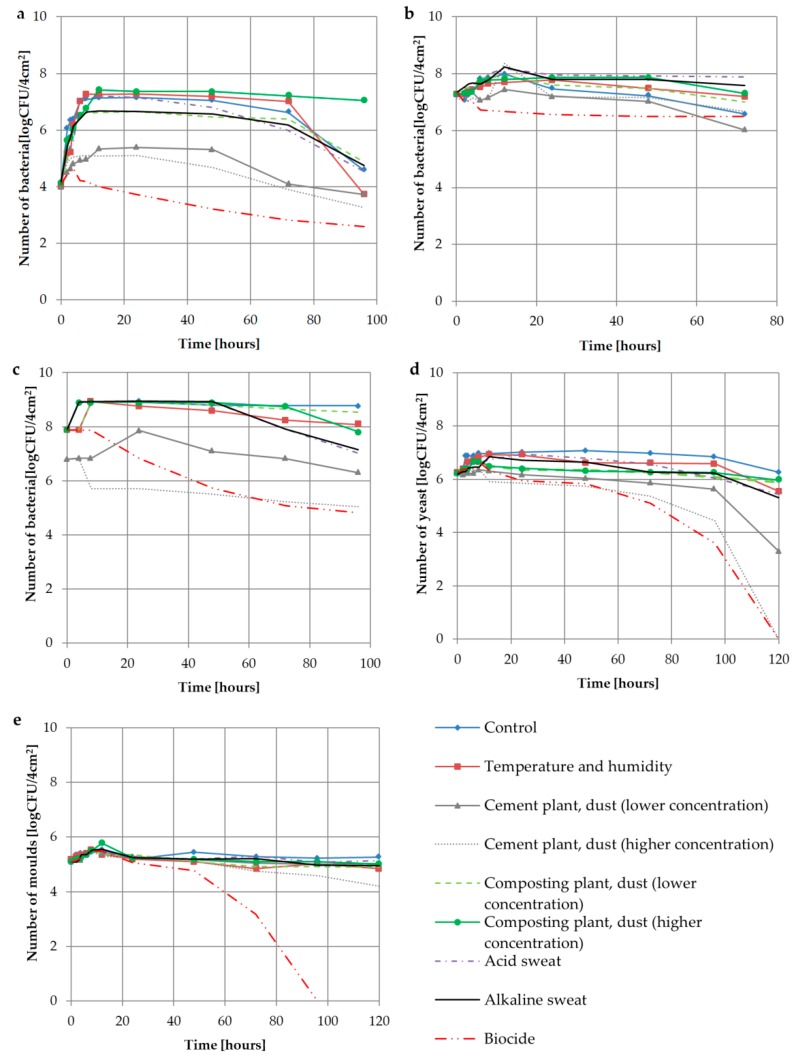
The dynamics of microbial survival on the filtering nonwoven during the incubation depending on the factors studied: (**a**) *B. subtilis*; (**b**) *S. aureus*; (**c**) *E. col*; (**d**) *C. albicans*; (**e**) *A. niger*.

The results of the correlations between microorganism numbers on filtering nonwovens and studied factors at individual incubation times are presented in Figure 2. Microorganism survival kinetics had a typical course with separate logarithmic, stationary and death growth phases. The deaths of all studied microorganisms were observed on filtering nonwovens with biocide right from the beginning of the incubation. It was also shown that dust from the cement dust at both low and high levels constituted an unfavourable factor for the growth of the studied microorganisms. We observed that *B. subtilis*, *E. coli* bacteria and *C. albicans* yeast died the fastest and to the greatest extent on the nonwovens with biocide and cement plant dust. The remaining microorganisms, *S. aureus* and *A. niger*, died much more slowly, with the exception of the variant containing biocide (Figure 2).

The analysis of the impact of the factors studied on microorganism growth parameters showed that the presence of cement dust at low concentrations on the filtering nonwovens lengthened the stationary phase for *E. coli* (λ_stat_ 5.72 h) and *B. subtilis* (λ_stat_ 32.64 h) compared to that of controls (*E. coli* λ_stat_ 4.69 h; *B. subtilis* λ_stat_ 22.28 h) (Table 4, I-control, II and IV dust from cement plant). At high concentrations, dust from a cement plant had a negative influence on the growth of all microorganisms studied. It shortened the stationary phase. On the other hand, the presence of dust from a composting plant on filtering nonwovens caused a lengthening of the stationary phase for *S. aureus* (λ_stat_ 7.21 h—low dust concentration and λ_stat_ 30.62 h—high dust concentration) and *E. coli* (λ_stat_ 8.0 h—high dust concentration) compared to that of control nonwovens (*S. aureus* λ_stat_ 4.21 h; *E. coli* λ_stat_ 4.69 h) (Table 4, I-control, V and VI dust from the composting plant).

We observed that addition of both acidic and alkaline sweat onto filtering nonwovens caused the shortening of the stationary phase compared to control nonwovens for all microorganisms, except for *E. coli.* The latter caused the lengthening of this phase (Table 4, I-control, VII–VIII acidic and alkaline sweat).

Changing temperature and RH conditions during the exploitation of the filtering nonwovens had a positive influence on the growth of *S. aureus* (λ_stat_ 10.34 h). Only for these bacteria, the lengthening of the stationary phase was ascertained compared to the control (λ_stat_ 4.21 h) (Table 4, I-control, II-changing conditions of temperature and RH).

The addition of biocide to the filtering nonwovens caused a shortening of the stationary phase of all tested microorganisms (λ_stat_ 2.61–4.13 h) and a reduction of cell increment (Y_max_ 0.07–0.57 log CFU/4 cm^2^) compared to the control nonwoven (λ_stat_ 4.21–46.77 h and Y_max_ 0.29–3.03 log CFU/4 cm^2^) (Table 4, I—control, IX—biocide). The use of biocide significantly shortened the time of biological hazard, especially in the case of *S. aureus*, *B. subtilis* bacteria and *C. albicans* yeast (up to 4 h), confirming the rational for using biocide-modified FFRs. There was a greater increase in the tested bacteria on the filtering nonwovens (Y_max_ 3.03 log CFU/4 cm^2^) than there was for fungi (Y_max_ 0.72 log CFU/4 cm^2^). Therefore, it can be concluded that the bacteria tested were a greater biological threat than the fungi (Table 4).

### 3.2. Study at Workplaces

#### 3.2.1. Analysis of Dust Fractions in the Air 

The results of particulate matter (PM) measurements at workplaces comprising the fractionation of measured dust are shown in Table 5.

Statistically significant differences in given dust fraction mass concentrations prior (sample X) and during work (sample Y) were determined for all of the analysed workplaces (Table 5). The highest mass concentration of dust prior to commencing work activities was observed in the cultivated field of farm 2 (*p* < 0.05), whilst the lowest was observed in both background measurements on farm 1 (1AX and 1BX). This tendency was observed for every dust fraction tested. During professional activities, higher dust concentrations were noted at workplaces on farm 1. However, these differences were not significant (*p* > 0.05) because of the high mass concentration variability confirmed by high standard deviation values. In measurements carried out prior to commencing professional activities at workplaces designated 1AX, 2AX, and 2BX, the dominant PM fraction had an aerodynamic diameter greater than 10 µm and it accounted for 44.8, 52.4 and 41.2 wt.% of total dust concentration, respectively. The second largest fraction had an aerodynamic diameter below 1 µm and it accounted for 37.1, 24.1 and 30.7 wt.% of total dust concentration, respectively. Reverse proportions were observed at the workplace labelled 1BX. In this case, the dominant PM fraction had an aerodynamic diameter below 1 µm (65.0 wt.%) while the second largest fraction had an aerodynamic diameter greater than 10 µm (19.8 wt.%). In all instances, PM with aerodynamic diameter between 1 and 10 µm had a negligible contribution to total dust concentration (0.2–3.2 wt.%) and PM with an aerodynamic diameter between 4 and 10 µm ranged between 13.2 and 24.9 wt.% For measurements made while the farmers worked, in all instances examined, the dominant PM fraction had an aerodynamic diameter greater than 10 µm and it accounted for over half of the total dust concentration (51.2–54.9 wt.%). The second largest fraction had an aerodynamic diameter below 1 µm, and it was the smallest in the case of the workplace marked 1AY (23.3 wt.%) and the largest for the workplace marked 2BY (33.2 wt.%). Similar to background measurements, the PM of aerodynamic diameter between 1 and 10 µm contributed negligibly to the total dust concentration (0.1–1.6 wt.%) and the PM of aerodynamic diameter between 4 and 10 µm ranged between 12.8 and 20.6 wt.%

#### 3.2.2. Microbiological Analysis of Air, Sedimented Dust and FFRs

An overall increase in air bacteria number was noted at all workplaces while carrying out harvest-related work (7.5 × 10^3^–7.2 × 10^4^ CFU/m^3^ of air) compared to a sample collected prior to commencing the work (5.4 × 10^3^–1.6 × 10^4^ CFU/m^3^ of air) (Table 6). A statistically significant (α = 0.05) higher overall bacteria number was demonstrated at the workplace of a combine harvester operator (1A) for air samples taken prior and during work. The increase of bacteria numbers in the air during harvest-related work may be related to an increased air dust concentration, confirmed by dust granulometric analysis at the workplaces examined (Table 5). The overall fungi numbers in the air at workplaces examined were similar, regardless of the sampling timing. No statistical differences were demonstrated (Table 6).

Overall bacteria numbers (3.1–3.3 × 10^6^ CFU/g) in sedimented dust samples collected at workplaces of different sites were 1–2 log higher than the overall fungi number (2.8 × 10^4^–1.2 × 10^5^ CFU/g) (Table 7). *Pseudomonas fluorescens* (4.5 × 10^6^ CFU/g) and actinomycetes 2.7 × 10^6^ CFU/g) dominated the dust samples examined. Tests of dust sedimented at the combine harvester workplace (A) showed that overall xerophilic fungi, mannitol-positive and mannitol-negative *Staphylococci* in dust sample I (dust sedimented on the combine harvester) was significantly higher (α = 0.05) than in dust sample II (dust collected behind the combine). Statistical analysis also demonstrated lower overall fungi numbers in dust sample III (dust sedimented in the indoor farm premises) at a tractor driver’s workplace (B) compared to dust sample I (dust on the combine) collected at the workplace of a combine harvester operator as well as lower overall xerophilic fungi numbers in sample II (combine harvester operator’s workplace) compared to sample III (tractor driver’s workplace) (Table 7).

**Figure 3:**
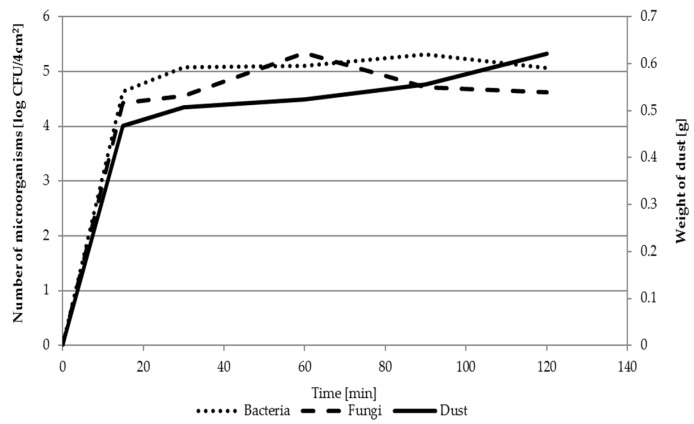
Dynamics of changes in the number of microorganisms and the amount of dust on the FFRs during their use at the combine operator’s workplaces.

The analysis of bacteria numbers on the FFRs at a combine harvester operator’s workplace demonstrated the greatest microorganism number increase on FFRs used for 30 min (bacteria) and 60 min (fungi). The greatest increase of dust mass was ascertained after 30 min of FFRs exploitation (circa 0.5 g). Later, the value increased slightly to 0.55–0.62 g after 120 min of FFRs use (Figure 3).

**Figure 4:**
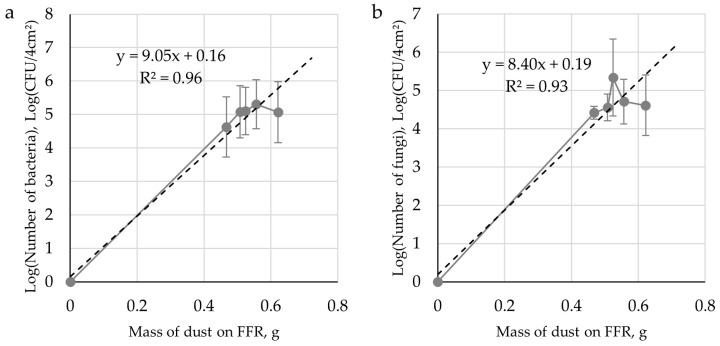
Logarithm dependence of the number of microorganisms and the mass of dust accumulated on FFRs (**a**) bacteria and (**b**) fungi.

We observed that the microorganism numbers on FFRs depended powerfully on the amount of dust retained on them. We confirmed this in modelling studies that showed a direct correlation between the deposited dust amount and microorganism growth. The R^2^ fit coefficient determined by linear regression equaled 0.96 for bacteria and 0.93 for fungi (Figure 4).

The time point at which microorganism numbers on the FFRs is the highest during its exploitation constitutes important information regarding the biological threat to workers. In studies conducted at the combine harvester operator’s workplace, this time was 30 min for bacteria and 60 min for fungi. An important parameter is also the growth of microorganisms during the use of FFRs, reflecting the level of biological threat. In this study, the bacterial cell increment was calculated as 4.94 log CFU/4 cm^2^, and for fungal, it was 4.90 log CFU/4 cm^2^.

## 4. Discussion

The survival of microorganisms on filtering materials used in FFRs depends on many factors, including microorganism type, exposure time, the presence and concentration of sedimented dust, humidity and presence of biocides within the material. In a previous study, it was shown that survival depended primarily on the type of microorganism and their physiological conformation (in the form of single vegetative cells, spores), bioaerosols and capacity of biofilm growth [26,39]. The significance of organic dust concentrations (e.g., plant biomass used in a combined heat and power station) was also ascertained. This modifies survival depending on microorganism type. It stimulates the growth of *E. coli*, inhibits that of *S. aureus* bacteria and *C. albicans* yeast, and has no effect on *A. niger* moulds [26]. In other studies, breathing simulation indicated that the level of air humidity inside the filtering material increased to RH = 80–90% after 7 min with temperatures to 29–30 °C. In such conditions, good microbial growth on filtering materials used for the protection of respiratory system was observed [20]. By contrast, no significant influence of filtering material type (melt-blown electret, spun-bonded, needle-punched calendared) used in the FFRs on microbial growth was found. High, comparable microorganism growth was established for all material types [37].

Current studies in a model system showed that material humidity (ensured by the presence of water and also of sweat, both acidic and alkaline) and the presence of dust from the composting plant determined high microorganism survival. Instead, dust from the cement plant, especially at high concentrations (60 mg/4 cm^2^ of nonwoven surface) and Sanitized biocide (TH2227, Sanitized AG, Switzerland, 4 wt.%) in the filtering material significantly limited the survival of *E. coli*, *S. aureus*, *B. subtilis*, *C. albicans* and *A. niger* microorganisms.

All microorganisms studied were characterized by good growth in variable humidity on filtering material corresponding to FFRs used in workplaces. Air humidity at RH = 100% inside filtering material, as well as during breaks from work (RH = 40%) when the material is stored at room temperature, stimulated microorganism growth. Current research showed that during the entire duration of use and storage of the FFRs, there were favourable conditions for microorganism growth. The results of studies of filtering materials, on which the formation of bacterial and fungal biofilms was observed at high humidity and dust pollution conditions, both in model systems and in the work environment at a plant biomass processing combined heat and power station, confirm these results [10,26,37].

We also observed that dust from the composting plant significantly increased microbial survival, especially that of bacteria (*E. coli*, *S. aureus*), causing the lengthening of the stationary phase compared to control samples without dust. Previous analyses of chemical composition and cytotoxicity of dust from the composting plant showed that it constituted an optimal environment for microorganism growth and, of all dust types examined, it displayed the lowest cytotoxic effect on the A-549 human adenocarcinoma lung epithelial adherent cell line [36]. The current study indicates that an increase to 37% of the concentration of dust from the composting plant supports the growth of all five types of microorganisms. The studies at workplaces in various categories of composting plants (facilities producing substrates for industrial cultivation of button mushrooms and facilities for processing biodegradable waste) indicate a high microbiological air and surface pollution and the presence of potential pathogens [40]. The development of these microorganisms on FFRs may constitute a substantial biological hazard. In the current study, dust from the cement plant elicited the opposite effect from that of dust from the composting plant. This may be due to its toxic character and chemical composition that does not support microorganism growth [36]. Dust from the cement plant particularly inhibited *E. coli, B. subtilis* and *C. albicans*.

Sanitized biocide was the factor that inhibited the growth of all microorganisms on filtering materials. Previous studies have shown that the use of this biocide for the modification of nonwovens in FFRs is an effective way to limit the growth of microorganisms; however, it depends on microorganism type, the kind of biocide and concentration as well as its carrier [30,31,41,42,43]. Sanitized biocide, containing quaternary ammonium salts, has been used in pilot studies to modify FFRs. It was found that its activity depends both on concentration (0.7–2.0%) and on the application method [40]. Present studies showed that material containing Sanitized at 4 wt.% and water-absorbing additive (EK-X EN52, Nippon/Ecotec, at 2 wt.%), produced by melt-blowing, was effective in reducing microbial growth, shortened the stationary phase of microbial growth (to 2–4 h, depending on the microorganism) compared to the control (4–47 h) and reduced the cell number increment by up to 5–6 logs.

Since model studies showed that organic dust may significantly increase microorganism survival on FFRs, environmental studies at farmers’ workplaces during harvest, characterised by one of the highest levels of dust pollution, were carried out as the next research stage [36]. Farmers are exposed to organic dusts of plant origin when carrying out various tasks (e.g., field collection, threshing and grain storage) [44,45,46].

The deposition site and retention time of airborne particles within the respiratory system varies widely depending on the aerodynamic size and shape of the particles [47]. Those two factors are among main determinants of the toxicity of inhaled particles. That is why the principle of particle size-selective thresholds in their occupational exposure limits (OELs) is commonly accepted worldwide. OELs may differ significantly depending on the region. There are considerable differences between EU community exposure limits, national exposure limits established individually by the Member States and those established in non-European countries. Moreover, OELs may vary depending on the specific type of dust. For organic dusts such as grain dust, 8 h OELs vary from 1 mg/m^3^ (Japan-JSOH) to 10 mg/m^3^ (Ireland, USA-OSHA and the United Kingdom).

Dust concentration values reported in our study were in almost all cases higher than 2 mg/m^3^, higher than corresponding OELs established in Poland where the measurements took place (2 mg/m^3^ for respirable fraction and 4 mg/m^3^ for inhalable fraction of organic dust of plant or animal origin). Moreover, they all exceed the suggested limit values specified in air quality guidelines of World Health Organization for airborne particulate matter that are equal to 0.025 mg/m^3^ for PM_2.5_ and 0.050 mg/m^3^ for PM_10_, respectively.

Several recent studies have provided information regarding PM concentration measurements at workplaces where airborne organic particles are present; however, overall occupational exposure and epidemiologic studies are still necessary to gain a complete understanding regarding their effects on the safety and well-being of workers. Current literature data on the PM concentrations resulting from grain harvesting are rather limited. It is primarily focused on ambient air measurements over areas neighbouring the fields where the crops are harvested [48]. Gutarowska et al. reported that elevated levels of dust resulting from rapid movement of the grain material through the air can depend on the array of activities performed by the workers along the entire harvesting process [36]. Our study shows that, not only the differences in harvesting activities can lead to diverse PM concentration levels, but also that equipment and machinery can be significant contributing factors in this regard. Furthermore, the results obtained within the study underline the importance of preventive and protective measures that should be undertaken in such work processes.

The dust in farmers’ working environments constitutes a major threat, as it is a source of microorganisms. The amount and type of dust correlates with microorganism numbers, as observed in environmental tests through the microbiological analysis of air, dust and FFRs.

The overall bacteria number in the air was shown to be 7.5 × 10^3^–7.2 × 10^4^ CFU/m^3^, and of fungi it was 5.7 × 10^3^–7.0 × 10^4^ CFU/m^3^. Such levels are similar to those reported in literature for sweet corn and soy harvest, herb and grain processing and grain storing workplaces, where fungal pollution levels were 10^3^–10^6^ CFU/m^3^ and bacterial levels 10^4^–10^6^ CFU/m^3^ [11,49,50,51]. Microbiological air pollution at examined farmers’ workplaces at harvest did not exceed the quantitative reference thresholds specified by the literature or those of the Polish Committee for the Highest Permissible Concentrations and Intensities of Noxious Agents in the Workplace, which is 1.0 × 10^5^ CFU/m^3^ for bacteria and 5.0 × 10^4^ CFU/m^3^ for fungi [52,53]. Only at one workplace (tractor driver, grain transport to the place of unloading) was this level exceeded (overall number of fungi 7.2 × 10^4^ CFU/m^3^).

Microbiological analysis of sedimented dust showed that bacteria constituted the main pollutant. Overall bacteria number equaled 3.1–3.3 × 10^6^ CFU/g, higher than the overall fungi number (2.8 × 10^4^–1.2 × 10^5^ CFU/g). These results were concordant with those of previous studies of dust samples collected from blower elements transporting freshly harvested wheat from the field to the silo [36]. Tests showed that actinomycetes and *Pseudomonas fluorescens* bacteria dominated in grain dust, with xerophilic fungi to a lesser extent.

Microbiological contamination of FFRs used at workplaces studied was very high: 10^5^ CFU/4 cm^2^ of filtering material, for both bacteria and fungi. In another study, Szulc et al. [10] determined a much lower level of microbiological contamination of FFRs at a combined heat and power station processing plant biomass (10^1^–10^2^ CFU/1 cm^2^). With the passing of FFRs use time at the workplace of a harvest combine operator, the number of bacteria increased by 5 logs after 30 min, and for fungi by 5 logs after 60 min. The weight of dust retained on the FFRs changed similarly: Initially, quickly up to 0.47 mg/FFRs in 30 min; later, more slowly up to 0.62 g/FFRs at 120 min of its use. The correlation between microorganism numbers and dust weight on FFRs during its use at 120 min was high (R^2^ = 0.93–0.96).

The results suggest that the problem of microbiological contamination of FFRs can be aggravated by unfavourable environmental conditions. The maim problem that was identified is high humidity content in the filtering material, contributing to the microbial growth. A simple solution to this problem would be to develop methods by which the reduction of the amount of moisture in these materials would be possible. Such solutions focusing on the modification of filtering materials with hydrophilic additives have already been experimentally investigated and have shown promising results in terms of comfort properties [54,55]. Another solution, confirmed by the presented results, is the use of nonwovens containing biocidal agents that can significantly reduce the number of microorganisms in filtering material. However, as long as such material solutions do not reach the market of respiratory protective devices, it is necessary to apply the rules for the proper use of equipment. In particular, a single-use-only FFRs should be used every time a high concentration of biologically contaminated dust is present. Moreover, FFRs should be replaced several times within one work shift, study results suggest that it should be even as often as once every hour.

## 5. Conclusions

The moisture content of the material (the presence of water as well as of acidic or alkaline sweat) and the presence of organic dust (in tests of dust from the composting plant) are the factors that determine the high survival rate of microorganisms on filtering materials used in FFRs. Test microorganisms *E. coli*, *S. aureus*, *B. subtilis*, *C. albicans* and *A. niger* were characterised by good growth in the conditions of variable humidity on the filtering material corresponding to the use and storage of FFRs at workplaces. This constitutes a serious threat when utilising reusable FFRs. We found that the Sanitized biocide used in the filtering material (4 wt.% concentration) was effective in reducing the growth of microorganisms, contributing to the shortening of the stationary growth phase and decreasing the cell number increment by up to 5–6 logs.

The tests at farmers’ workplaces revealed high dust concentrations. In almost all instances, it was higher than OELs and WHO limits for airborne particulate matter. Microbiological pollution at tested farmers’ workplaces at harvest was high but did not exceed the quantitative reference thresholds specified by the literature. Microorganism numbers in the sedimented dust at workplaces studied were also high. Actinomycetes and *Pseudomonas fluorescens* as well as xerophilic fungi constituted the main contaminants. There was also a high microbiological contamination at workplaces of FFRs at the level of 10^5^ CFU/4 cm^2^ of filtering material. The correlation between microorganism numbers and the weight of dust on the FFRs during its exploitation for 120 min was high (R^2^ = 0.93–0.96). The application of the Gompertz equation in model studies to describe the parameters of microorganism growth on filtering material and to calculate cell increment and the time of safe use of FFRs in field studies allowed for the determination of the basic threat during FFRs use at a combine harvester operator’s workplace. This threat was the presence of organic dust with high bacteria and fungi contamination. Because of the high level of dust pollution during harvest, it is recommended that single-use FFRs should be replaced several times during on a work shift. In this work environment, the use of reusable FFRs may pose a microbiological hazard to employee health.

## Figures and Tables

**Table 1:** Characteristics of filtering nonwovens.

No.	Nonwoven Type	Thickness * (mm)	Surface Mass ** (g/m^2^)	Paraffin Oil Mist Penetration *** (%)	Air Flow Resistance **** (Pa)
1.	electret Polypropylene melt-blown nonwovens (PPQ)	1.73 ± 0.13	75.8 ± 9.9	7.33 ± 1.91	200 ± 31
2.	composite electret Polypropylene melt-blown nonwovens (CPPQ)	2.97 ± 0.18	135.3 ± 6.5	1.50 ± 0.17	361 ± 16

* Tested according to ISO 9073-2:1995 standard [32]; ** Tested according to EN 29073-1:1992 standard [33]; *** Tested according to EN 13274-7:2002 standard [34] at 20 mg/m^3^ and 95 L/min air flow rate; **** Tested according to EN 13274-3:2001 standard [35] at 95 L/min air flow rate.

**Table 2:** Dust content in the nonwoven samples.

Mass of Dust Deposited in the Nonwoven (mg/sample)			
Dust from the Composting Plant		Dust from the Cement Plant	
2 min (deposition time)	4 min (deposition time)	2 min (deposition time)	4 min (deposition time)
X: 24.6	X: 51.2	X: 33.6	X: 60.6
SD: 10.4	SD:24.7	SD: 10,9	SD:15.9

Sample—79 cm^2^; X—mean; SD—standard deviation.

**Table 3:** Workplace characteristics.

No.	Study Location	Type of Work Performed at Workstation	Type of Sample Taken at Workstation	Microclimate Conditions
1.	Cultivated field (2.7 ha), indoor working premises, Odolin, Lodz voivodeship, Poland (DMS: 52°10′44.271′′ N 19°36′27.669′′ E)	**A**—combine harvester operator—open cab without glazing (wheat harvesting and pouring grain onto a trailer with the use of a feeder)	Air: **X** prior, **Y** during working the fields	T: 385 RH: 27.8 W: 0.87
			Dust: **I** dust sedimented on the combine, **II** dust gathered behind the combine (from the rear end of the machine)	
		**B**—tractor driver (grain transport to the site of collection and storage on the farm premises)	Air: **X** prior, **Y** during working inside the premises	T: 35.6 RH: 30.9 W: 0.1
			Dust: **III** sedimented on the premises	
2.	Cultivated field (5.2 ha), indoor working premises, Wola Miłkowska, Lodz voivodeship, Poland (DMS: 51°48′42.452′′ N 18°36′15.331′′ E)	**A**—combine harvester operator—closed cab with glazing (wheat harvesting and pouring grain onto a trailer with the use of a feeder)	Air: **X** prior, **Y** during working the fields	T: 33.2 RH: 34.9 W: 0.22
			Dust: **I** dust sedimented on the combine, **II** dust gathered behind the combine (from the rear end of the machine)	
		**B**—tractor driver (grain transport to the site of collection and storage on the working premises, straw baling)	Air: **X** prior, **Y** during working inside the premises	T: 28.8 RH: 42 W: 0.15
			Dust: **III** sedimented on the premises	

T—Temperature [°C], RH—Relative humidity [%], W—Air flow velocity [m/s]; W, E, N, S—geographical location.

**Table 4:** The influence of factors on the growth parameters of microorganisms on the filtering nonwoven.

Microorganism	Growth Parameters	Factors								
		I	II	III	IV	V	VI	VII	VIII	IX
*B. subtilis*	λ_stat_ (hours)	22.28	18.17	32.64	24.53	19.01	10.86	14.57	18.37	2.61
	t_stat_ (hours)	5.93	7.15	8.08	2.95	5.71	6.22	6.10	7.43	3.98
	Y_max_ (log CFU/4 cm^2^)	3.03	3.24	1.28	1.01	2.40	3.03	2.93	2.50	0.57
*E. coli*	λ_stat_ (hours)	4.69	1,00	5.72	3.42	1.00	8.00	19.99	20.22	3.38
	t_stat_ (hours)	8.19	8.53	6.49	3.01	11.89	4.18	4.19	4.31	5.83
	Y_max_ (log CFU/4 cm^2^)	0.95	2.68	0.66	0.22	0.99	0.63	0.96	1.01	0.20
*S. aureus*	λ_stat_ (hours)	4.21	10.34	2.45	0.34	7.21	30.62	1.36	3.77	nb
	t_stat_ (hours)	8.54	17.87	9.80	11.76	6.66	9.16	18.51	8.88	nb
	Y_max_ (log CFU/4 cm^2^)	0.92	0.52	0.16	1.39	0.61	0.54	0.72	0.44	0.21
*A. niger*	λ_stat_ (hours)	43.79	1.94	2.22	10.69	3.17	1.18	19.56	17.52	3.29
	t_stat_ (hours)	4.81	5.31	7.44	4.51	4.92	12.98	9.15	9.89	9.13
	Y_max_ (log CFU/4 cm^2^)	0.29	0.21	0.31	0.16	0.19	0.60	0.43	0.48	0.40
*C. albicans*	λ_stat_ (hours)	46.77	7.81	1.18	1.91	3.56	0.59	16.76	4.92	4.13
	t_stat_ (hours)	4.18	12.41	11.35	8.37	4.66	8.68	9.24	10.63	4.57
	Y_max_ (log CFU/4 cm^2^)	0.72	0.70	0.19	0.21	0.18	0.61	0.58	0.49	0.07

nb—not studied, bacteria decay after the inoculum was applied to the nonwoven, in which case the growth of bacteria *S.aureus* was not observed; I control-nonwoven PPQ; II temperature and humidity; III and IV cement plant, dust (lower and higher concentration); V and VI composting plant, dust (lower and higher concentration); VII acidic sweat; VIII alkaline sweat; IX biocide. Growth parameters: λ_stat_—stationary phase duration; t_stat_—stationary phase beginning time; Y_max_—cell increment.

**Table 5:** Airborne dust mass concentrations at workplaces.

Workplace	Sample Type	Airborne Dust Mass Concentrations Corresponding to Particle Size Fractions (mg/m^3^)				
		PM_1_	PM_2.5_	PM_4_	PM_10_	PM_total_
1A	X	M: 0.09 ^a^	M: 0.09 ^a^	M: 0.09 ^a^	M: 0.13 ^b^	M: 0.23 ^b^
		SD: 0.15	SD: 0.16	SD: 0.16	SD: 0.27	SD: 0.60
	Y	M: 2.47 ^a^^,^*	M: 2.50 ^a,^*	M: 2.63 ^a,^*	M: 4.79 ^a,^*	M: 10.62 ^a,^*
		SD: 3.72	SD: 3.75	SD: 3.92	SD: 6.98	SD: 15.90
1B	X	M: 0.07 ^a^	M: 0.07 ^a^	M: 0.07 ^a^	M: 0.08 ^a^	M: 0.10 ^a^
		SD: 0.02	SD: 0.02	SD: 0.02	SD: 0.04	SD: 0.09
	Y	M: 2.53 ^a,^*	M: 2.56 ^a,^*	M: 2.70 ^a,^*	M: 4.76 ^a,^*	M: 10.25 ^a,^*
		SD: 1.98	SD: 2.00	SD: 2.10	SD: 3.65	SD: 7.87
2A	X	M: 0.79 ^c^	M: 0.80 ^c^	M: 0.84 ^c^	M: 1.57 ^d^	M: 3.29 ^d^
		SD: 0.32	SD: 0.32	SD: 0.34	SD: 0.68	SD: 1.41
	Y	M: 1.93 ^a,^*	M: 1.94 ^a,^*	M: 2.03 ^a,^*	M: 3.52 ^a,^*	M: 7.22 ^a,^*
		SD: 1.65	SD: 1.66	SD: 1.73	SD: 2.83	SD: 5.88
2B	X	M: 0.16 ^b^	M: 0.16 ^b^	M: 0.18 ^b^	M: 0.31 ^c^	M: 0.52 ^c^
		SD: 0.17	SD: 0.18	SD: 0.20	SD: 0.40	SD: 0.74
	Y	M: 2.01 ^a,^*	M: 2.02 ^a,^*	M: 2.05 ^a,^*	M: 2.83 ^a,^*	M: 6.05 ^a,^*
		SD: 3.73	SD: 3.74	SD: 3.77	SD: 4.76	SD: 9.77

M—mean, SD—standard deviation, *—statistically significant differences between the mass concentration of a given dust fraction before work (X) and during their duration (Y) for a given work station (test, α = 0.05); a, b, c, d—statistically significant differences in concentration of a given dust fraction for various work stations (within the column for the same type of sample) are marked with different letters (Anova, α = 0.05, Tukey’s test, α = 0.05).

**Table 6:** Microbiological contamination of air at workplaces.

Workplace	Sample Type	Microorganism Number (CFU/m^3^)	
		Bacteria	Fungi
1A	X	M: 6.3 × 10^3^	M: 1.7 × 10^4^
		SD: 1.2 × 10^3^	SD: 6.3 × 10^3^
	Y	M: 2.5 × 10^4^ *	M: 6.9 × 10^3^
		SD: 9.2 × 10^3^	SD: 2.5 × 10^3^
1B	X	M: 1.6 × 10^4^	M: 4.5 × 10^3^
		SD: 4.9 × 10^3^	SD: 7.4 × 10^2^
	Y	M: 7.2 × 10^4^	M: 7.2 × 10^4^
		SD: 5.9 × 10^4^	SD: 5.9 × 10^4^
2A	X	M: 5.4 × 10^3^	M: 6.6 × 10^3^
		SD: 3.2 × 10^3^	SD: 5.8 × 10^3^
	Y	M: 7.5 × 10^3^	M: 5.7 × 10^3^
		SD: 1.8 × 10^3^	SD: 2.0 × 10^3^
2B	X	M: 8.0 × 10^3^	M: 3.9 × 10^3^
		SD: 3.4 × 10^3^	SD: 1.3 × 10^3^
	Y	M: 3.4 × 10^4^	M: 6.7 × 10^3^
		SD: 9.9 × 10^3^	SD: 3.8 × 10^3^

M: mean; SD: standard deviation; * are significantly different in the number of microorganisms in the air samples before (X) and during working (Y) (test ANOVA, α = 0.05).

**Table 7:** The number of microorganisms in dust samples settled at workplaces.

Marked Microorganisms	Microorganism Number (CFU/g)		
	A/I	A/II	B/III
Bacteria	M: 3.1 × 10^6 a^	M: 3.3 × 10^6 a^	M: 3.1 × 10^6 a^
	SD: 1.8 × 10^6^	SD: 5.7 × 10^5^	SD: 5.4 × 10^5^
Actinomycetes	M: 2.7 × 10^6 a^	M: 2.7 × 10^6 a^	M: 2.0 × 10^6 a^
	SD: 1.3 × 10^6^	SD: 2.0 × 10^6^	SD: 5.2 × 10^5^
*Staphylococci* spp.*	M: 7.4 × 10^4 a^	M: 3.8 × 10^4 b^	M: 6.7 × 10^4 a,b^
	SD: 1.3 × 10^4^	SD: 1.9 × 10^4^	SD: 3.5 × 10^4^
*Pseudomonas fluorescens*	M: 4.4 × 10^6 a^	M: 4.5 × 10^6 a^	M: 4.1 × 10^6 a^
	SD: 1.4 × 10^6^	SD: 1.3 × 10^6^	SD: 4.7 × 10^5^
Fungi	M: 1.2 × 10^5 a^	M: 2.8 × 10^4 b^	M: 8.1 × 10^4 b^
	SD: 3.7 × 10^4^	SD: 1.5 × 10^4^	SD: 3.5 × 10^4^
Xerophilic Fungi	M: 2.0 × 10^5 a^	M: 3.4 × 10^4 b^	M: 1.1 × 10^5 a^
	SD: 8.1 × 10^4^	SD: 1.4 × 10^4^	SD: 4.7 × 10^4^

M: mean; SD: standard deviation; a, b: means with the same letter in the same row are not significantly different (test ANOVA, α = 0,05); * Mannitol-Positive and Mannitol-Negative *Staphylococci* spp.
